# Drug Metabolism and Toxicological Mechanisms

**DOI:** 10.3390/toxics13060464

**Published:** 2025-05-31

**Authors:** Qi Wang, Youbo Zhang, An Zhu

**Affiliations:** 1 Department of Toxicology, School of Public Health, Peking University, Beijing 100191, China; 2 State Key Laboratory of Natural and Biomimetic Drugs, School of Pharmaceutical Sciences, Peking University, Beijing 100191, China; 3 Key Laboratory of Gastrointestinal Cancer (Fujian Medical University), Ministry of Education, Fuzhou 350108, China

The metabolism of drugs and xenobiotics is a cornerstone of pharmacology and toxicology, governing the efficacy, safety, and environmental impact of therapeutic agents [1]. Drug metabolism involves enzymatic transformations that convert lipophilic compounds into water-soluble metabolites for excretion, primarily mediated by cytochrome P450 (CYP) enzymes, UDP-glucuronosyltransferases (UGTs), and transporters [2]. These processes occur in two phases, functionalization (Phase I) and conjugation (Phase II), followed by excretion [3]. While metabolism is essential for detoxification, it can also generate reactive intermediates that contribute to organ damage, carcinogenesis, or immune-mediated toxicity [4]. Understanding these dual roles—detoxification versus toxification—is critical for drug development and personalized medicine.

Variability in drug metabolism, driven by genetic polymorphisms (e.g., CYP2D6 and CYP2C19), age, disease states, and environmental factors (e.g., diet and pollutants), complicates toxicity prediction. For instance, slow metabolizers of codeine due to CYP2D6 deficiencies risk opioid toxicity from unchecked conversion to morphine, while ultra-rapid metabolizers face overdose risks [5]. Similarly, drug–drug interactions (DDIs) involving enzyme induction or inhibition—such as grapefruit juice’s inhibition of CYP3A4—can alter therapeutic outcomes [6,7]. These examples underscore the need for precision toxicology frameworks that account for individual and population-level variability.

Historically, toxicology relied on observational and empirical methods, but modern approaches integrate mechanistic insights from molecular biology, omics technologies (genomics, proteomics, and metabolomics), and computational models. Despite progress, challenges persist in predicting idiosyncratic reactions, rare adverse events, and long-term environmental impacts. Traditional animal models often fail to replicate human-specific metabolic pathways, spurring innovations like organ-on-a-chip systems, 3D organoids, and humanized mouse models [8]. Meanwhile, regulatory agencies increasingly demand mechanistic data to complement traditional safety assessments, reflecting a paradigm shift toward “adversity pathways” and quantitative risk modeling.

This Special Issue addresses these complexities by showcasing 12 peer-reviewed articles (11 original research papers and 1 review) that span molecular mechanisms, clinical toxicology, preclinical studies, and regulatory innovation. Contributions explore themes such as enzyme polymorphisms, environmental toxicants, and AI-driven toxicity prediction. Collectively, they highlight three transformative trends: (1) the integration of multi-omics to unravel metabolic networks, (2) the adoption of advanced in vitro and in silico models to reduce animal testing, and (3) the application of big data to bridge gaps between preclinical findings and clinical outcomes. In summary, this Special Issue reflects the dynamic evolution of drug metabolism research, emphasizing mechanistic clarity, technological innovation, and translational relevance.

## 1. An Overview of Published Articles

The study by Tian et al. (contribution 1) systematically elucidated the hepatotoxic mechanisms of mesaconitine (MA) through in vivo zebrafish models, revealing dose-dependent impairment of hepatic development characterized by reduced liver size, neutrophil infiltration, and ROS accumulation. Transcriptomic profiling and molecular validation demonstrated that MA exposure triggered endoplasmic reticulum stress through oxidative damage, activating the unfolded protein response (UPR) via upregulation of key chaperones. Prolonged UPR activation drives hepatocyte apoptosis through caspase-dependent pathways. These findings established a ROS-UPR–apoptosis axis as the molecular basis for MA-induced liver injury, bridging the gap between *Aconitum* alkaloid exposure and hepatic dysfunction. The use of transgenic zebrafish lines allowed real-time visualization of liver injury progression, neutrophil infiltration, and apoptosis dynamics, highlighting the unique advantages of zebrafish in mechanistic toxicology. This study not only advanced zebrafish-based toxicology assessment methodologies but also provided essential safety data for the clinical application of traditional medicines.

The study by Trakulsrichai et al. (contribution 2) systematically described the epidemiological features, clinical manifestations, therapeutic strategies, and prognostic factors of ethephon poisoning through a retrospective analysis of clinical data from 252 patients in the Ramathibodi Poison Center database. The study found that 21.4% of individuals exposed to ethephon exhibited no apparent symptoms, and gastrointestinal symptoms constituted the most frequently reported clinical manifestations at 55.6% in all cases. Furthermore, the mortality rate remained relatively low, representing 0.8% of the total ethephon-exposed population. However, advanced-age intentional ingestion and early neurological symptoms such as impaired consciousness were significantly associated with adverse outcomes. As a study with a large reported sample size, this research comprehensively elucidated the clinical characteristics of ethephon poisoning, addressing a critical lack of human data for this field. Furthermore, by integrating epidemiological and toxicological perspectives, the study validated the World Health Organization classification of ethephon as slightly hazardous, supporting its safety assessment. Future investigations can explore the metabolic mechanisms of ethephon and its association with atypical cholinergic manifestations. The authors not only systematically delineated the clinical profile of ethephon poisoning but also advanced precision medicine through prognostic analysis. This work established a benchmark for pesticide toxicity management and toxicological research, serving as a valuable reference for both clinical practice and public health initiatives.

The study by Lin et al. (contribution 3) investigated the mechanisms underlying the effects of the environmental endocrine disruptor bisphenol S (BPS) on hepatic lipid metabolism, revealing the biological processes through which BPS promotes lipid accumulation via oxidative stress-mediated molecular pathways. The research team utilized two hepatocellular cell models, HepG2 and SK-Hep-1, combined with cell viability assays, oxidative stress indicator analyses, and measurement of lipid metabolism-related gene and protein expression to systematically characterize the metabolic disturbances in hepatocytes exposed to BPS. The results demonstrated that BPS significantly suppressed the expression of PPARα and CPT1B, key regulators of fatty acid oxidation, while upregulating the expression of SREBP1C and FASN, genes associated with lipid synthesis. Oxidative stress further exacerbated lipid droplet deposition, ultimately leading to hepatic lipid metabolism imbalance. By systematically dissecting the molecular mechanisms through which BPS disrupts lipid synthesis and degradation pathways via oxidative stress in hepatic cell models, this study identified the critical regulatory role of the PPARα/SREBP1C-FASN axis. Additionally, the identified targets, such as PPARα and SREBP1C, provide new directions for developing interventions targeting lipid metabolism disorders. Through multi-timepoint and multi-concentration gradient exposure experiments, the authors dynamically elucidated the dose–response relationship and time-dependent toxicity of BPS, significantly enhancing the credibility of the conclusions.

The study by Gonçalves et al. (contribution 4) systematically investigated the regulatory effects of ethanol intake on paracetamol, also named acetaminophen (APAP)-induced hepatotoxicity, using a C57BL/6 mouse model. The findings revealed that acute ethanol (AE) exacerbated APAP-induced liver injury by elevating CYP2E1 activity, TBARS, and GSSG, manifested as expanded necrotic areas, increased ALT/AST levels, and upregulated inflammatory cytokines. In contrast, chronic ethanol (CE) significantly mitigated liver damage by enhancing UGT1A1 gene expression, restoring glutathione homeostasis, and reducing carbonylated protein levels, with hepatic pathological features resembling those of the control group. Proteomic profiling further demonstrated that the CE group clustered with the control group in protein expression patterns, whereas the AE group aligned with the APAP group, suggesting that chronic ethanol consumption may confer protective effects through metabolic adaptation mechanisms. By integrating histopathological, oxidative stress, gene expression, and proteomic analyses, the study elucidated the critical regulatory role of the UGT1A1/CYP2E1 axis, providing molecular mechanistic evidence for understanding ethanol–drug metabolic interactions. Additionally, the identified UGT1A1 upregulation mechanism highlighted novel therapeutic targets for hepatoprotective strategies. The authors dynamically dissected molecular networks underlying liver injury through proteomics, enhancing the credibility of conclusions. This work established a paradigm for research on drug–alcohol interactions, offering significant guidance for rational medication practices and toxicological risk assessment.

The study by Yin et al. (contribution 5) systematically investigated the acute toxicity and cardiotoxicity of the natural phenolic acid compound protocatechuic aldehyde (PCA) in juvenile zebrafish. Through exposure experiments with different concentration gradients (50–80 µg/mL) of PCA, the study found that high concentrations (70 and 80 µg/mL) of PCA caused severe malformations in zebrafish larvae, including spinal curvature, pericardial edema, significant reduction in locomotor activity, and cardiac structural abnormalities and functional dysfunction through phenotypic observation, behavioral analysis, cardiac function assessment, and histopathological evaluation. Further analysis using network pharmacology predictions and RT-PCR validation revealed that the inhibition of genes such as *PIK3CA*, *PARP1*, and *GSK3β* mediated cardiotoxicity through regulation of the PI3K/AKT/GSK3β signaling pathway. This study provides a comprehensive evaluation of PCA’s toxic effects in a zebrafish model, offering important theoretical insights for safety assessments of PCA-containing drugs and the development of low-toxicity pharmaceuticals.

The study by Yang et al. (contribution 6) investigated the nephrotoxicity and mechanisms of *Cassiae semen* aqueous extracts (CSAEs) through a 28-day repeated-dose experiment in rats. The study revealed that CSAEs induced dose-dependent increases in serum creatinine and blood urea nitrogen levels, accompanied by morphological alterations in rat kidneys. Using molecular docking and experimental validation, the authors identified that key CSAE components, namely, obtusifolin, aurantio-obtusin, and obtusin, exhibited strong binding affinity to F-actin, ROCK1, and Rac1, with the RhoA-ROCK pathway identified as the regulatory mechanism mediating CSAE nephrotoxicity. Immunofluorescence staining confirmed disrupted renal cell membranes and brush borders, establishing F-actin as a primary toxicity target. Further experiments demonstrated that CSAEs dose-dependently suppressed mRNA expression of RhoA-ROCK pathway genes such as *ROCK1* and *cofilin* and reduced GTP-RhoA protein and phosphorylated ROCK/cofilin levels, leading to actin depolymerization, cytoskeletal destabilization, and subsequent nephrotoxicity. This study linked *Cassiae semen* toxicity to aberrant actin cytoskeleton remodeling, offering novel insights into the mechanisms of natural product-induced nephrotoxicity.

The study by Kim et al. (contribution 7) evaluated the risk of optic neuropathy (ON) and visual impairment induced by ethambutol in tuberculosis treatment in a retrospective cohort analysis of 204,598 ethambutol users in the Korean NHIS database from 2015 to 2021, tracked to 2022. The findings revealed that 2.6% of ethambutol-treated patients developed ON, with risk factors including female sex, older age, higher cumulative ethambutol doses, longer treatment duration, and systemic comorbidities such as diabetes, hypertension, hyperlipidemia, kidney and liver diseases, and malnutrition. Notably, concomitant isoniazid use demonstrated a protective effect with an OR value of 0.78, mediated by shortened ethambutol exposure, while deficiencies in vitamins B1, B6, and B12 exacerbated ethambutol-induced neurotoxicity. Despite limitations inherent to its retrospective design, the study provides critical guidance for monitoring and preventive strategies in ethambutol-treated patients, emphasizing the importance of personalized risk assessment and interventions. Overall, this research advances the understanding of ethambutol-associated ON and its interactions with isoniazid, systemic comorbidities, and nutritional status, and underscores the necessity of mitigating ON risks to optimize tuberculosis treatment protocols.

The study by Wan et al. (contribution 8) investigated how rutaecarpine affected APAP-induced liver toxicity. Mouse and liver cell models demonstrated that rutaecarpine aggravates liver injury, as evidenced by significantly elevated serum ALT and AST levels, extensive hepatic necrosis with substantial parenchymal damage on histopathological examination, and notable upregulation of inflammatory cytokines, including IL-6 and IL-1β. Mechanistic studies revealed that rutaecarpine induced CYP1A2 expression at both mRNA and protein levels, accelerating APAP metabolism to its toxic metabolite NAPQI. These effects were reversed by α-Naphthoflavone, a CYP1A2 inhibitor, confirming the enzyme’s critical role in accelerating APAP metabolism. The study reveals the risks of combining rutaecarpine with APAP, providing critical insights for clinical drug safety and the rational use of traditional Chinese medicine (TCM) with synthetic drugs.

The study by Gao et al. (contribution 9) conducted a proteomic analysis on 20 laryngeal squamous cell carcinoma patient samples, uncovering the significant role of ribosome biogenesis pathways in tumor metastasis. Their study demonstrated a clear relationship between lymph node metastasis and the overexpression of ribosomal proteins (RPS10 and RPL24), heightened activity in protein synthesis pathways, and disrupted cell adhesion mechanisms, creating a network that promotes metastatic spread. In vitro experiments further revealed that the RNA polymerase I inhibitor CX-5461 exhibited anti-metastatic properties at non-cytotoxic concentrations by reversing epithelial–mesenchymal transition and reducing the levels of ribosomal proteins, thereby suppressing tumor cell invasion and migration. Despite the known DNA-damaging effects of CX-5461 posing challenges to its clinical use, both the authors and reviewers acknowledge the potential of developing therapeutic strategies with inhibitory activity against ribosome biogenesis pathways based on optimizing drug design and comprehensive safety evaluations. This study not only deepens our understanding of ribosomal dysregulation in LSCC metastasis but also emphasizes the significance of the pathology-based grouping approach in proteomic analysis in identifying potential cancer targets and providing personalized treatment recommendations.

The study by Wu et al. (contribution 10) conducted epigenetic, proteomic, and metabolomic analyses on the human trophoblast cell line HTR-8/SVneo, revealing the reproductive toxicity effects induced by oridonin. The study found that the Wnt/β-catenin signaling pathway, tight junction, thiamine metabolism, and amino acid degradation pathways were all involved in the toxic effects of oridonin on HTR-8/SVneo cells. Further in vitro experiments demonstrated that the toxic effects involved elevated intracellular Ca²⁺ levels, oxidative stress, the occurrence of mitochondrial dysfunction, DNA damage, and abnormal expression of molecules related to the Wnt/β-catenin signaling pathway and tight junction. These findings confirmed that the inhibition of the Wnt/β-catenin signaling pathway and disruption of the tight junction were key mechanisms underlying oridonin-induced cytotoxicity. Despite oridonin’s significant pharmacological potential in anti-inflammatory, anti-tumor, and antibacterial applications, its reproductive toxicity poses a critical limitation to its clinical use. By deeply elucidating the toxic mechanisms of oridonin, it is possible to provide a theoretical basis for optimizing its clinical application strategies, thereby maximizing its therapeutic potential while ensuring safety. This study not only provides a deeper understanding of oridonin’s reproductive toxicity mechanisms through a multi-omics approach but also underscores the value of integrated multi-omics analysis in identifying toxicity targets. It establishes a novel research framework for evaluating the safety of active compounds in TCM.

The study by Bian et al. (contribution 11) investigated the biochemical and toxicological changes following insulin overdose using a rat model, aiming to identify specific molecular markers for forensic diagnosis. Here, 20 IU/kg insulin aspart induced severe hypoglycemia, convulsions, and metabolic disturbances, including reduced glucose and glycogen levels, along with elevated lactate. Key findings revealed potassium redistribution due to activated Na^+^-K^+^-ATPase and stimulation of the PI3K-AKT pathway in skeletal muscle, promoting GLUT4 translocation, suggesting that detecting GLUT4 and Na^+^-K^+^-ATPase proteins on the skeletal muscle cell membrane can provide valuable auxiliary diagnostic information for forensic identification of insulin overdose. The study proposes the ratio of insulin to C-peptide as a forensic marker for identifying exogenous insulin overdose, addressing the challenges in postmortem insulin detection. This work bridges clinical insights with forensic applications, offering valuable tools for postmortem diagnosis, particularly useful when physical evidence of injection is absent.

The study by Mohammed et al. (contribution 12) conducted a comprehensive review focusing on the toxicity mechanisms, clinical presentations, and diagnostic and treatment approaches for methanol poisoning, emphasizing the importance of preventive measures. Methanol is metabolized into formaldehyde and formic acid, leading to severe metabolic acidosis and multiorgan damage, particularly affecting the central nervous system and vision. The article highlighted that clinical manifestations vary by age and exposure type. Diagnosis relies on clinical evaluation, laboratory tests, and advanced techniques like gas chromatography. Methanol poisoning results in severe metabolic acidosis and multiorgan damage due to the accumulation of formic acid, leading to significant morbidity and mortality. Effective clinical management includes timely administration of antidotes like fomepizole or ethanol and hemodialysis for severe cases. This article provides a comprehensive perspective on understanding methanol poisoning, from its pathogenesis to clinical management, offering valuable guidance for healthcare providers.

## 2. Conclusions

The articles in this Special Issue collectively advance our understanding of drug metabolism and toxicity, offering novel insights and tools to address longstanding challenges. A study investigated CYP450 isoforms to reveal disparities in drug response and toxicity risks. Several papers dissect mechanisms of organ-specific toxicity, such as hepatotoxicity from acetaminophen and nephrotoxicity from *Cassiae* semen aqueous extracts. Environmental toxicology is also well represented, with studies on the impacts of pollutants on metabolic pathways. Emerging technologies are another highlight. Mechanistic toxicology has seen significant progress. Papers elucidating mitochondrial dysfunction, oxidative stress, and immune-mediated injury provide frameworks for predicting organ-specific toxicity. Such mechanisms offer biomarkers for early toxicity detection and targets for protective interventions.

The rise of alternatives to animal experimentation marks a paradigm shift in toxicology. Zebrafish (*Danio rerio*) and *Caenorhabditis elegans* (*C. elegans*) have become invaluable models for high-throughput drug toxicity screening and mechanistic studies [9]. These tools not only enhance predictive accuracy but also align with global efforts to promote the 3Rs (Replacement, Reduction, and Refinement) in toxicology. Their unique biological features, combined with advanced genetic and molecular techniques, enable rapid hypothesis testing while reducing reliance on mammalian systems. Zebrafish have gained prominence due to their genetic tractability, optical transparency during early development, and high genetic similarity to humans (87%). These attributes facilitate real-time observation of organogenesis and physiological responses to toxicants. For instance, zebrafish mutants like space cadet—which exhibit aberrant escape responses due to disrupted Mauthner cell connectivity—provide insights into neurotoxicant effects on behavior [10]. Meanwhile, *C. elegans* offers a tractable system to study metabolic perturbations, as seen in research linking mitochondrial dysfunction to drug-induced oxidative stress [11]. Its short lifecycle of 3 days, fully mapped genome, and conserved metabolic pathways make it ideal for high-throughput screens. These models not only reduce reliance on mammals but also enable rapid hypothesis testing in drug metabolism and toxicology [12].

TCMs present unique challenges due to their complex compositions and potential for herb–drug interactions. Metabolomics has emerged as a powerful tool to unravel TCM toxicity, as demonstrated in studies. Similarly, UPLC-Q-TOF/MS-based analyses of toxicity identified detoxification mechanisms, highlighting the synergy between modern technology and traditional knowledge [13]. Such research aligns with global efforts to standardize TCM safety assessments while preserving their therapeutic potential.

Some research methods are not covered in this Special Issue, and these techniques are also worthy of application in drug toxicology and metabolism studies. The integration of advanced technologies is a milestone. Organ-on-a-chip platforms replicate human tissue interactions, enabling real-time monitoring of metabolite kinetics and toxicity [14]. In parallel, AI models trained on multi-omics datasets demonstrate remarkable accuracy in predicting idiosyncratic reactions, bridging gaps between in vitro assays and clinical outcomes [15]. These tools are poised to revolutionize preclinical testing, reducing reliance on animal models and accelerating drug discovery. Additionally, the rise of biologics and gene therapies demands new metabolic paradigms, as traditional small-molecule pathways may not apply. Regulatory science must also evolve. While mechanistic data are increasingly valued, standardization of novel methodologies such as organoids and AI models is needed for regulatory acceptance. Global collaboration, as seen in the FDA Emerging Technology Program, can harmonize guidelines and foster innovation [16].

Looking ahead, the field must embrace interdisciplinary collaboration. Integrating pharmacogenomics, environmental science, and computational toxicology will yield holistic models of metabolic networks. Public health initiatives, such as biobanking and global toxicity databases, can enhance predictive power and equity in drug safety. In closing, this Special Issue not only catalogs current achievements but also charts a course for future inquiry. By prioritizing mechanistic depth, technological innovation, and translational impact, the toxicology community can mitigate adverse drug reactions, safeguard environmental health, and usher in an era of precision medicine.

## Data Availability

Data sharing is not applicable (only appropriate if no new data is generated or the article describes entirely theoretical research).
